# Psychological Impact of COVID-19 on People with Pre-Existing Chronic Disease

**DOI:** 10.3390/ijerph18115972

**Published:** 2021-06-02

**Authors:** Michael Owusu Budu, Emily J. Rugel, Rochelle Nocos, Koon Teo, Sumathy Rangarajan, Scott A. Lear

**Affiliations:** 1 Faculty of Health Sciences, Simon Fraser University, Burnaby, BC V5A 1S6, Canada; emily.rugel@sydney.edu.au (E.J.R.); rochelle_nocos@sfu.ca (R.N.); slear@providencehealth.bc.ca (S.A.L.); 2 Community Health Research Team, St. Paul’s Hospital, Vancouver, BC V6Z 1Y6, Canada; 3 Westmead Applied Research Centre (WARC), The University of Sydney, 2145 Sydney, Australia; 4 Population Health Research Institute, Hamilton, ON L8L 2X2, Canada; koon.teo@phri.ca (K.T.); sumathy.rangarajan@phri.ca (S.R.)

**Keywords:** chronic illness, anxiety, depression, COVID-19

## Abstract

The COVID-19 pandemic has caused an increase in anxiety and depression levels across broad populations. While anyone can be infected by the virus, the presence of certain chronic diseases has been shown to exacerbate the severity of the infection. There is a likelihood that knowledge of this information may lead to negative psychological impacts among people with chronic illness. We hypothesized that the pandemic has resulted in increased levels of anxiety and depression symptoms among people with chronic illness. We recruited 540 participants from the ongoing Prospective Urban and Rural Epidemiology (PURE) study in British Columbia, Canada. Participants were asked to fill out an online survey that included the Hospital Anxiety Depression Scale (HADS) to assess anxiety and depression symptoms. We tested our hypothesis using bivariate and multivariable linear regression models. Out of 540 participants, 15% showed symptoms of anxiety and 17% reported symptoms of depression. We found no significant associations between having a pre-existing chronic illness and reporting higher levels of anxiety or depression symptoms during COVID-19. Our results do not support the hypothesis that having a chronic illness is associated with greater anxiety or depression symptoms during the COVID-19 pandemic. Our results were similar to one study but in contrast with other studies that found a positive association between the presence of chronic illness and developing anxiety or depression during this pandemic.

## 1. Introduction

The coronavirus disease (COVID-19) has rapidly spread across 223 countries and territories since its initial identification in December 2019. By the end of January 2021, close to 100 million cases and more than 2 million deaths had been confirmed worldwide [1]. In response to this global health crisis, numerous policies have been implemented by governmental and public-health agencies around the world to help reduce the spread of COVID-19.

Evidence from research on previous outbreaks has shown that factors such as confinement (due to self-isolation or quarantine mandates), income reduction, and uncertainties surrounding outbreaks are associated with increased anxiety and depression levels [2,3,4,5]. For instance, Hawryluck and colleagues conducted a cross-sectional survey in 2004 to examine the psychological effects of confinement among 129 quarantined individuals during the SARS outbreak in Toronto, Canada. The researchers found depressive symptoms in 32% of the respondents. They also reported that participants with the lowest annual household income (income < CAD 40,000) had the highest mean CES-D score of 18.3 ± 15.4, which indicates the presence of mild clinical depression [3]. 

Studies about the psychological impact of COVID-19 have been conducted in multiple countries, with many reporting associations between the pandemic and increased levels of anxiety and depression [6,7,8,9,10,11]. A national cross-sectional survey was conducted in Italy to determine the effects of the COVID-19 lockdown on mental health and sleep disturbance. The researchers used the Generalized Anxiety Disorder-2 scale (GAD-2) and Patient Health Questionnaire-2 (PHQ-2) to determine the presence of anxiety and depression and found anxiety symptoms in 23% of the participants and depression in 25% [6].

Distinct psychological impacts of the COVID-19 pandemic are not only experienced among older adults but also among adolescents. Multiple studies have reported increased levels of anxiety, depression, and stress among adolescents during this pandemic [12,13]. While anyone can be infected with COVID-19, older adults, those who are immuno-compromised, and people with pre-existing chronic medical conditions are at increased risk of severe illness [14,15,16,17,18]. Chronic medical conditions with the most consistent evidence of adverse impact on COVID-19 include cancer, COPD, heart conditions (heart failure, coronary artery disease, or cardiomyopathies), obesity, and diabetes mellitus [19]. Given the potential for chronic illness to exacerbate adverse consequences, such as higher risk of hospitalization and death, individuals with these diseases might be expected to experience additional concerns related to COVID-19. 

Our research, therefore, aimed to explore the association between pre-existing chronic illness and mental wellbeing among participants in Vancouver, British Columbia (BC), Canada during this pandemic. We hypothesized that people with chronic medical conditions would report higher levels of anxiety and depression symptoms during the COVID-19 pandemic than those without these conditions. 

## 2. Materials and Methods

### 2.1. Study Population

Individuals participating in the Prospective Urban and Rural Epidemiology (PURE) cohort—a community-based, 18-year observational study that examines how urbanization affects health behaviours, primary risk factors, and cardiovascular disease (CVD) [20] —were recruited. Although PURE is a global study, we only recruited participants residing in BC for this study. Taking advantage of electronic communication methods to reduce participant risks and enhance enrollment, we initially sent a study invitation via email to all active PURE participants in BC (*n* = 2432). Within two weeks, those who had not responded to the email were telephoned by the research centre to explain the study and invite them to participate. Individuals who agreed to participate were asked to sign a consent and participate in the survey. Consenting was done either by phone or via email. There was no in-person contact or procedure throughout the study to support the physical-distancing measures in place across the province of BC. Our survey consisted of questions on demographics, COVID-19, and psychological symptoms captured via the Qualtrics survey platform. This study was approved by the Providence Health Care and Simon Fraser University Research Ethics Boards.

### 2.2. Anxiety and Depression

Symptoms of anxiety and depression were measured with the Hospital Anxiety and Depression Scale (HADS), which has been shown to be reliable and valid for assessing the level of anxiety and depression in large-scale population surveys [21]. HADS consists of two self-report scales: one that assesses anxiety and one that assesses depression. Each scale has seven questions (14 in total), with scores for each question ranging on a Likert-scale measure of 0–3. The highest possible score for each of the anxiety and depression subscales is 21, with a total score of seven or less regarded as the absence of symptoms, a score between 8–10 regarded as mild, 11–14 as moderate, and 15–21 as severe. The HADS questionnaire has been validated in many languages, countries, and settings, including general practice and community sites [22]

The survey was conducted from April to June 2020, which was the initial peak period of the pandemic in BC. The increase in COVID-19 cases during this period forced BC’s Provincial Health Authority to declare a public health emergency and also to issue orders to cancel public gatherings and cease operations of personal-service establishments [23]. In addition, the Federal government advised all residents to stay at home and restricted all non-essential travel outside Canada during this time period [24].

### 2.3. Chronic Illness

Data on chronic illness were abstracted from the participant research file from the most recent PURE follow-up visit. Follow-ups for the PURE study in general occur every three years, and we are currently in our 12th year of follow-up. For most people, the most recent BMI and chronic disease follow-up was between 2018 and 2020. We limited our definition to participants with diagnoses of cancer, diabetes, obesity, COPD, or any of a number of forms of heart disease (comprising coronary artery disease, heart failure, and cardiomyopathies) for the purpose of this study. These conditions were selected because findings of their impact on the severity of COVID-19 have been consistent across multiple studies [14,16,17,18]. During this assessment, body mass index (BMI) was determined through measured height and weight. Participants with a BMI ≤ 24.9 kg/m^2^ were classified as having normal weight, BMI of 25–29.9 kg/m^2^ as overweight, and BMI > 30 kg/m^2^ as obese. Individuals with a BMI > 30 kg/m^2^ were also classified as having a chronic illness in this study. This is due to the fact that numerous studies and health institutions recognize obesity as a chronic illness [25,26,27].

### 2.4. Confounders

Potential confounders were selected a priori based on the existing literature [8,11,28] and comprised age, sex, household size, and education. Confounders such as age, sex, and education were obtained from the participant research file. Data on household size were obtained through the online survey via the question “How many people live with you in your household?”, with response options of “0”, “1–3”, and “4 or more” people.

### 2.5. Mediation

We hypothesized that social interaction was a mediator of the association between chronic illness and higher levels of anxiety or depression symptoms. Data on social interactions were obtained by asking participants two questions: (1) “How many people outside your household have you interacted with in person in the past week”; and (2) “How many people outside your household have you had a verbal conversation with either online or on the telephone in the past week?”. In order to test this hypothesis, we conducted mediation analysis using the zMediation approach [29]. This approach considers the statistical significance of the association between explanatory variables (chronic illness) and outcomes (anxiety or depression), explanatory variables (chronic illness) and mediators (social interactions), and then mediators (social interactions) and outcomes (anxiety or depression). However, our results showed no significant association between the explanatory variable (chronic illness) and mediator (social interactions) in either bivariate or multivariable analyses. As a result, we did not proceed to the next steps of the mediation analysis. Social interactions were therefore not included in the final model.

### 2.6. Statistical Analysis

We initially performed descriptive analyses for all variables. Means and standard deviations were used to describe continuous values, while numbers and percentages were used for categorical values. Participants were then grouped based on their chronic disease status (yes or no) and a one-way ANOVA or chi-square test (Fisher test when required) was used to determine how the two groups (participants with or without chronic disease) compare in relation to the various variables. Similar analyses were conducted between the two groups and our outcome variables. We then developed separate bivariate and multivariable linear regression models to test the association between chronic illness and anxiety, as well as between chronic illness and depression, as measured via HADS. Our final multivariable model included continuous measures of anxiety and depression as independent outcomes, with chronic illness as the primary explanatory variable, and all of the confounders reported above. We also carried out a sensitivity analysis to examine the relationship without including obesity as a chronic disease, and our findings did not change (data available upon request). All analyses were carried out using SAS statistical software, version 9.4 of the SAS system for Windows (SAS Institute Inc., Cary, NC, USA, 2013).

## 3. Results

### 3.1. Sample Characteristics

A total of 650 PURE subjects consented to participate. One hundred and ten (110) participants were excluded from the study subsequently as a result of missing data from the HADS questionnaire or on confounders, including household size, social interactions, and whether participants were working outside of the home during the pandemic. The final sample, therefore, included 540 participants aged 34–70, of which 58% were women. The mean age of participants was 52 years, with 28% aged 34–46 years, 52% aged 47–59 years, and 20% aged 60 years and above (see Table 1). With regards to education, 83% had completed post-secondary education. 

### 3.2. Chronic Illness

One hundred and forty-seven (147) participants (27%) reported having one of the selected chronic conditions. Within this group, 85% of participants reported having only one condition, with the remainder having two or more. The most common condition was obesity, among 67% of participants; with heart failure being the least common at only 1%. 

### 3.3. Psychological Disorders

- Anxiety

Participants’ mean anxiety score was 4.50 ± 3.21 out of a maximum total of 21. Mean anxiety scores were significantly higher in female participants between 34–46 years. (see Table 2). Based on the standard cut-off score, 15% of participants showed symptoms of anxiety, either mild (*n* = 61) or moderate (*n* = 24); there were no cases of severe anxiety recorded. The number of anxiety cases among people without chronic illness (*n* = 65) was three times higher than those with pre-existing chronic illness (*n* = 20). Our analysis showed a non-significant difference in the likelihood of developing anxiety between participants with chronic illness and those without chronic illness (*p* = 0.43) (see Table 3). In the bivariate linear regression model, we found a negative non-significant association between chronic illness and anxiety levels (β = −0.15; 95% CI = −0.74, 0.43; *p*-value = 0.61). In the multivariable linear regression model, our results showed a positive non-significant association between having chronic illness and reporting higher levels of anxiety symptoms (β = 0.05; 95% CI = −0.52, 0.62; *p*-value = 0.85) (see Table 4).

- Depression

Participants’ mean depression score was 4.24 ± 3.21. Seventeen percent (17%) of participants reported symptoms of depression. Of those, 68 participants had mild and 26 had moderate symptoms. No participant reported symptoms that would be classified as severe. Among these participants, 70.2% (*n* = 66) of them had no pre-existing chronic illness. We found no significant difference in the likelihood of developing depression in relation to the presence or absence of chronic illness (*p* = 0.52) (see Table 3). The results of the bivariate linear regression analysis between chronic illness and depression showed a positive non-significant association (β = 0.25; 95% CI = −0.37, 0.86; *p*-value = 0.43). Similarly, after adjustment, we found a positive non-significant association between having a pre-existing chronic illness and reporting higher levels of depressive symptoms (β = 0.35; 95% CI = −0.25, 0.96; *p*-value = 0.25) (see Table 4).

## 4. Discussion

This study investigated whether there was an association between pre-existing chronic illness and increased symptoms of anxiety and depression during COVID-19. Data were collected during the initial phase of severe lockdown in BC. Among participants with chronic conditions, 14% of them showed symptoms of anxiety, while 19% had symptoms of depression. Although anxiety and depression cases were higher in people without chronic illness, our analyses showed no significant association between the presence or absence of chronic illness and either outcome. We also found that social interaction was not a significant mediator between chronic illness and either outcome. 

Reports from the Canadian Community Health Survey indicate 2.6% of the general population report symptoms of anxiety and 4.7% of depression [30]. A potential explanation for our finding of higher rates of both symptoms may have been as a result of the current COVID-19 pandemic. Evidence from various studies has shown a significant association between the pandemic and increased levels of anxiety and depression [6,7,8,9,10,11]. 

The lack of significant association in our results could be attributed to the various mental health support systems initiated by the health system. Our findings are similar to results from a study conducted by Louvardi and colleagues who also found no significant association between having chronic illness and developing anxiety (*p* = 0.098) or depression (*p* = 0.052) [31], but contrast with studies by other researchers who reported positive associations between chronic illness and both anxiety and depression during this pandemic [6,9,32,33]. One potential reason for this difference in results may be due to how chronic disease was defined in each study. While we limited our definition to people with either cancer, COPD, diabetes, obesity, or heart conditions (heart failure, coronary artery disease, or cardiomyopathies), the other studies had no such restrictions. There is therefore a likelihood that they found an association due to the other chronic diseases included in their study.

### Strengths and Limitations

Our study has several important strengths and limitations. First, because participants were selected from an ongoing longitudinal study, variables such as body mass index and the presence of chronic illness were recorded prior to the pandemic and also before anxiety and depressive symptoms were assessed by the research team. Participants’ height and weight were measured by the team and later used to calculate their BMI. Generalizing these results to the overall population should be done with caution because our study sample was not fully representative of the broader population. Although the PURE study is community-based, participants were not randomly selected, nor were the participants of this study. As a result, our findings may only be applicable to the specific populations represented by our participants. For example, our population comprised mainly highly educated, middle-aged men and women (52%), in contrast to the 19.3% in BC as of the 2016 census [34]. In addition, because participants of this study were self-selected, the possibility of self-selection bias cannot be ruled out. We adjusted for many important factors, some of which had not been considered as potential confounders in previous studies (e.g., household size), but we were lacking data on other confounders that may be important, such as chronic stress and household income. Future research that incorporates these variables would be beneficial. In addition, some of our participants may have developed chronic disease during the period between our most recent PURE study follow-up and when they completed the survey for this study. Furthermore, our study did not consider the severity of participants’ chronic illness, which may have had an impact on our findings. Finally, because our study was cross-sectional, there was no pre-pandemic data to compare with our results.

## 5. Conclusions

In summary, our results did not support our hypothesis that COVID-19 is related to higher levels of anxiety or depression symptoms among people with chronic illness as compared to those without. We also found that interacting with people either in person or online during this pandemic was not significantly related to levels of anxiety or depression among individuals with chronic disease. The percentage of people with anxiety or depression in our study was higher than that of the general population. Future research should focus on comparing levels of anxiety and depression over time to further examine this causal relationship.

## Figures and Tables

**Table 1:** Descriptive analysis of the sample (*n* = 540).

Variables	Total, *N* (%)	Chronic Disease		*p*-Value
		Yes, *n* (%)	No, *n* (%)	
Age (years), M ± SD	52 ± 8.50			*p* < 0.001 *
Sex				
Male	228	65 (29)	163 (71)	0.63
BMI				
Normal Weight	239 (45)	27 (11)	212 (89)	
Overweight	202 (37)	21 (10)	181 (90)	*p* < 0.0001 *
Obesity	99 (18)	99 (100)	0 (0)	
Education				
High School or less	90	27 (30)	63 (70)	0.52
Chronic illness				
Yes	147	147 (100)	0 (0)	
Social Distancing				
Yes	528	140 (27)	388 (73)	0.02 *
Self-Isolation				
Yes	294	83 (28)	211 (72)	0.62
Number of people living with participant in a household				
0	32 (6)	10 (31)	22 (69)	
1–3 people	440 (81)	121 (28)	319 (72)	0.68
4 and above	68 (13)	16 (24)	52 (76)	
Interactions in person (number of people in the past week)				
0	16 (3)	5 (31)	11 (69)	
1	25 (5)	9 (36)	16 (64)	
2	39 (7)	9 (23)	30 (77)	
3	45 (8)	16 (36)	29 (64)	0.79
4	46 (9)	13 (28)	33 (72)	
5	55 (10)	13 (24)	42 (76)	
6–9	145 (27)	38 (26)	107 (74)	
10–14	91 (17)	21 (23)	70 (77)	
15+	78 (14)	23 (29)	55 (71)	
Interactions online/on telephone (number of people in the past week)				
0	1 (0.2)	0 (0)	1 (100)	
1	14 (3)	3 (21)	11 (79)	
2	23 (4)	9 (39)	14 (61)	
3	32 (6)	7 (22)	25 (78)	0.81
4	28 (5)	10 (36)	18 (64)	
5	37 (6.8)	10 (27)	27 (73)	
6–9	137 (25)	40 (29)	97 (71)	
10–14	94 (17)	25 (27)	69 (73)	
15+	174 (32)	43 (25)	131 (75)	

*N* = total number of participants in each variable. *n* = number of participants under each characteristic based on the presence or absence of pre-existing chronic disease. * indicates significant association between variable and chronic illness (*p*-value < 0.05).

**Table 2:** Description of the samples stratified by mean anxiety and depression score.

		Anxiety		Depression	
	*N*	Mean ± SD	*p*-Value	Mean ± SD	*p*-Value
Age (years)					
34–46 years	151	5.30 ± 3.36		4.64 ± 3.51	
47–59 years	279	4.35 ± 3.30		4.19 ± 3.25	
60+ years	110	3.78 ± 2.51	*p* < 0.001 *	3.79 ± 2.56	0.30
Sex					
Male	228	3.72 ± 2.77		4.03 ± 2.89	
Female	312	5.07 ± 3.39	*p* < 0.001 *	4.39 ± 3.42	0.20
BMI					
Normal Weight	239	4.62 ± 3.37		3.97 ± 3.17	
Overweight	202	4.36 ± 3.13	0.76	4.24 ± 3.13	0.08
Obesity	99	4.49 ± 3.01		4.86 ± 3.42	
Education					
High School or less	90	4.12 ± 2.72	0.42	3.83 ± 3.05	0.18
Post-Secondary	450	4.58 ± 3.30		4.32 ± 3.24	
Chronic illness					
Yes	147	4.39 ± 3.02	0.88	4.41 ± 3.25	0.40
Social Distancing					
Yes	528	4.49 ± 3.22	0.46	4.22 ± 3.22	0.28
Self-Isolation					
Yes	294	4.56 ± 3.19	0.47	4.43 ± 3.28	0.13
Number of people living with participant in a household					
0	32	3.72 ± 2.87		3.56 ± 2.12	
1–3 people	440	4.43 ± 3.14	0.08	4.26 ± 3.30	0.67
4 and above	68	5.32 ± 3.68		4.37 ± 3.02	
Interactions in person (number of people in the past week)					
0	16	4.38 ± 3.81		4.31 ± 3.38	
1	25	4.72 ± 3.39		4.84 ± 3.76	
2	39	4.56 ± 3.47		4.64 ± 3.50	
3	45	5.04 ± 3.30		5.00 ± 3.04	
4	46	4.50 ± 3.10	0.89	4.46 ± 3.60	0.22
5	55	4.42 ± 2.92		4.20 ± 2.80	
6–9	145	4.58 ± 3.15		4.31 ± 3.14	
10–14	91	4.15 ± 3.39		3.89 ± 3.27	
15+	78	4.42 ± 3.13		3.54 ± 2.99	
Interactions online/on telephone (number of people in the past week)					
0	1	8.00 ± 0.00		12.00 ± 0.00	
1	14	4.00 ± 3.92		3.07 ± 2.64	
2	23	4.22 ± 3.72		4.09 ± 3.65	
3	32	4.47 ± 3.35		4.53 ± 3.26	
4	28	3.75 ± 2.44		4.14 ± 2.82	
5	37	4.92 ± 3.31	0.78	4.62 ± 3.90	0.70
6–9	137	4.59 ± 3.06		4.31 ± 3.20	
10–14	94	4.55 ± 3.01		4.14 ± 3.02	
15+	174	4.49 ± 3.40		4.18 ± 3.18	

*N* = total number of participants under each variable. SD = standard deviation. * indicates significant findings (*p*-value < 0.05).

**Table 3:** Description of anxiety and depression results stratified by pre-existing chronic illness.

Chronic Illness *n* (%)		No Chronic Illness *n* (%)	*p*-Value
Anxiety	20 (23.5)	65 (76.5)	0.43
Depression	28 (29.8)	66 (70.2)	0.52

**Table 4:** Results from regression analyses between chronic illness and either outcome.

Depression				Anxiety		
	β	95% CI	*p*-Value	β	95% CI	*p*-Value
Crude						
Chronic illness	0.25	−0.37 0.86	0.86	−0.15	−0.74 0.43	0.61
Adjusted						
Chronic illness	0.35	−0.25 0.96	0.25	0.05	−0.52 0.62	0.85

Adjusted for age, sex, education and household size. All *p*-values are 2-sided. Covariates: Social distancing, self-isolation, interaction in person, interaction online/on telephone.

## Data Availability

Data used for this study is available on request from the corresponding author. The data is not publicly available due to ethical and privacy concerns.
